# Effects of Seed-Applied Biofertilizers on Rhizosphere Biodiversity and Growth of Common Wheat (*Triticum aestivum* L.) in the Field

**DOI:** 10.3389/fpls.2020.00072

**Published:** 2020-02-26

**Authors:** Cristian Dal Cortivo, Manuel Ferrari, Giovanna Visioli, Marta Lauro, Flavio Fornasier, Giuseppe Barion, Anna Panozzo, Teofilo Vamerali

**Affiliations:** ^1^ Department of Agronomy, Food, Natural Resources, Animals and the Environment, University of Padua, Legnaro-Padua, Italy; ^2^ Department of Chemistry, Life Sciences and Environmental Sustainability, University of Parma, Parma, Italy; ^3^ Research Centre for Viticulture and Enology, Council for Agricultural Research and Analysis of Agricultural Economics (CREA), Gorizia, Italy

**Keywords:** plant growth-promoting rhizobacteria, vesicular arbuscular mycorrhizae, soil bacterial biodiversity, 16S rDNA sequencing, gluten composition, sustainable agriculture

## Abstract

In order to reduce chemical fertilization and improve the sustainability of common wheat (*Triticum aestivum* L.) cultivation, maintaining at the same time high production and quality standards, this study investigated the effects of three commercial biofertilizers on rhizosphere bacterial biomass, biodiversity and enzymatic activity, and on plant growth and grain yield in a field trial. The wheat seeds were inoculated with the following aiding microrganisms: (i) a bacterial consortium (*Azospirillum* spp. + *Azoarcus* spp. + *Azorhizobium* spp.); and two mycorrhizal fungal-bacterial consortia, *viz*. (ii) *Rhizophagus irregularis* + *Azotobacter vinelandii*, and (iii) *R. irregularis + Bacillus megaterium* + *Frateuria aurantia*, and comparisons were made with noninoculated controls. We demonstrate that all the biofertilizers significantly enhanced plant growth and nitrogen accumulation during stem elongation and heading, but this was translated into only small grain yield gains (+1%–4% vs controls). The total gluten content of the flour was not affected, but in general biofertilization significantly upregulated two high-quality protein subunits, i.e., the 81 kDa high-molecular-weight glutenin subunit and the 43.6 kDa low-molecular-weight glutenin subunit. These effects were associated with increases in the rhizosphere microbial biomass and the activity of enzymes such as β-glucosidase, α-mannosidase, β-mannosidase, and xylosidase, which are involved in organic matter decomposition, particularly when *Rhizophagus irregularis* was included as inoculant. No changes in microbial biodiversity were observed. Our results suggest that seed-applied biofertilizers may be effectively exploited in sustainable wheat cultivation without altering the biodiversity of the resident microbiome, but attention should be paid to the composition of the microbial consortia in order to maximize their benefits in crop cultivation.

## Introduction

The use of microbial inoculants is of strategic interest for their potential to replace chemical fertilizers and pesticides in agricultural systems, and improve environmental sustainability.

Plant-aiding microorganisms, often referred to as plant growth-promoting rhizobacteria (PGPR) (Gupta et al., 2015) and arbuscular mycorrhizal fungi (AMF) (Igiehon and Babalola, 2017), interact with plants roots (Hayat et al., 2010) by enhancing growth, mineral nutrition, drought tolerance, and disease resistance (Nadeem et al., 2013).

Bacteria can beneficially contribute to plant growth *via* N_2_-fixation and solubilization of low mobile nutrients. Biological N_2_-fixation is carried out by various symbiotic and nonsymbiotic bacteria (Shridhar, 2012).

Symbiotic PGPR fix atmospheric N_2_ mainly within plant roots, with many genera involved, such as *Rhizobium*, *Sinorhizobium*, *Bradyrhizobium*, *Mesorhizobium*, and *Azorhizobium*. The latter can enter plant roots intercellularly and colonize the xylem of wheat, rice, corn, and other nonlegume crops without forming real symbiotic structures (Cocking, 2003), although *Azorhizobium caulinodans* is known to form both stem and root nodules in *Sesbania rostrata* (Robertson et al., 1995).

Nonsymbiotic N_2_ fixation is carried out by free-living diazotrophic bacteria, such as *Azospirillum*, *Azoarcus*, *Azotobacter, Burkholderia, Gluconacetobacter, Clostridium,* and *Pseudomonas* (Bhattacharyya and Jha, 2012; Singh, 2018). The absence of symbiosis with plants supports their common use in biofertilizers formulation. These bacteria can improve the uptake efficiency of nitrogen in many crops, thanks to the nitrogenase activity and soil N mineralization (Chauhan et al., 2015). In addition, *Azotobacter* and *Azospirillum* stimulate root hair formation, and lateral and adventitious root initiation through hormonal (auxins) exchange (Vejan et al., 2016; Zeffa et al., 2019).

Some PGPR are also known as phosphate- and potassium-solubilizing bacteria through rhizosphere acidification (Afzal and Bano, 2008; Meena et al., 2014). Among these, *Bacillus megaterium* and *Frateuria aurantia* were reported as efficient P- and K-mobilizing bacteria, respectively, thus being potentially exploitable in crop cultivation (Subhashini, 2014; Ghaffari et al., 2018).

AMF are nonpathogenic fungi living in symbioses with roots of a large number of spontaneous and cultivated plants, supplying them with mineral nutrients and water, particularly in natural environments (Solaiman and Mickan, 2014). Fostering AMF-plant symbiosis through inoculation, can significantly improve nutrients accumulation, the plant physiological processes and biomass accumulation (Mitra et al., 2019), besides root growth promotion and abiotic stresses mitigation (Begum et al., 2019).

However, many biotic and abiotic factors may affect the ability of plant-aiding microorganisms to successfully colonize the rhizosphere (Ahmad et al., 2011), and hence impact on these effects (Kloepper and Beauchamp, 1992; Castro-Sowinski et al., 2007).

In a context of environmental protection and increased demand for chemical-free food products, increasing numbers of commercial biofertilizers have come onto the market in recent years, containing either single or associated PGPR strains or mycorrhizal fungi (Owen et al., 2015).

There is considerable evidence for the positive effects of PGPR on plant growth under controlled conditions, and their often ineffectiveness in the open field (Dobbelaere et al., 2003; Dal Cortivo et al., 2017), possibly due to the use of strains unable to adequately colonize plant roots and/or to compete with the resident rhizobiome (Lugtenberg and Dekkers, 1999; Bloemberg and Lugtenberg, 2001; Kamilova et al., 2005). Aiding microorganisms need to be introduced into agroecosystems in sufficient quantities to efficiently colonize plant roots, as this step is crucial for their success (Rao, 1993; Bishnoi, 2015). Following biofertilization, it can be expected positive or negative competition with the indigenous bacteria population, or no interaction, depending on survival strategies (Schwieger and Tebbe, 2000; Bacilio-Jimenez et al., 2001; Brimecombe et al., 2007). In this regard, essential bacterial behaviors include tolerance to nutrient- or water-limited conditions, affinity for root exudates, and competition with the resident rhizobacteria through the secretion of antibiotics (Paul and Nair, 2008; Nadeem et al., 2013; Kaur et al., 2017). Little information is as yet available on the effects of PGPR-AMF consortia introduced into the resident bacterial community structure. Effective use of biofertilizers therefore rests on a better understanding of their effects on soil microbial communities, and hence of the role they play in soil biodiversity and plant health (Roesti et al., 2006; Hart et al., 2017).

Previous studies have shown the positive effects of PGPR on plant productivity, particularly under stress conditions, suggesting their potential role in a climate change scenario where extreme events, such as floods and droughts, occur with greater frequency in cultivated land (Egamberdieva and Adesemoye, 2016). However, little is so far known of their effects on the quality of cereal grains.

Our previous investigations in open fields documented the ability of a N-fixing bacteria consortium (i.e., *Azospirillum* + *Azoarcus + Azorhizobium*), when applied as foliar spraying inoculum during tillering, to improve root growth and N accumulation in common wheat (Dal Cortivo et al., 2017). Similarly, a AMF-bacteria consortium of *Rhizophagus irregularis* + *Azotobacter vinelandii* was found to enhance root growth and mineral uptake in this crop (Dal Cortivo et al., 2018).

In this framework, the current multidisciplinary study investigated a spectrum of effects of different commercial biofertilizers (consisting of PGPRs alone or in association with AMFs) on rhizosphere enzymatic processes, microbial biomass, and biodiversity (using high-throughput next-generation sequencing—NGS), and on growth, grain yield and quality (gluten content and composition, not previously investigated) in common wheat. The aim was to gather information on the potential advantages of their use as seed inoculums instead of postemergence foliar spraying on a widely-cultivated crop and on the mechanisms involved, and to assess safety issues with respect to their interaction with the resident rhizobiome.

## Materials and Methods

### Biofertilizers

The following three biofertilizers were applied to fungicide-free seeds of common wheat (*Triticum aestivum* L.) immediately before sowing: (i) TN: TripleN^®^ (Mapleton Agri Biotec, Mapleton, Australia), at 0.02 g kg^-1^ of seeds, containing three PGPRs *Azorhizobium* spp., *Azoarcus* spp., and *Azospirillum* spp. (1×10^10^ CFU g^-1^); (ii) R-N: Rhizosum N^®^ (Biosum Technology, Madrid, Spain), at 0.25 g kg^-1^ of seeds, containing the AMF *Rhizophagus irregularis* (previously known as *Glomus intraradices*) (2% w/w) and *Azotobacter vinelandii* (1×10^10^ CFU g^-1^); and (iii) R-PK: Rhizosum PK^®^ (Biosum Technology, Madrid, Spain), at 0.375 g kg^-1^ of seeds, containing the AMF *Rhizophagus irregularis* (2% w/w) together with *Bacillus megaterium* (0.66 ×10^10^ CFU g^-1^) and *Frateuria aurantia* (0.33 ×10^10^ CFU g^-1^). The inoculum doses followed the manufacturers' recommendations. The freeze-dried inoculum was mixed with ultrapure water (10 ml kg^-1^ of seeds) in order to enhance its adherence to the seed surface, and 5 ml kg^-1^ of seeds of Delfan Plus (Tradecorp, Madrid, Spain), which contains amino acids for early bacterial activation.

### Experimental Design

The experiment was carried out in open field at the experimental farm of the University of Padua (Legnaro, Padua, NE Italy). The site has a deep, silty-loam soil (fulvi-calcaric-cambisol; USDA classification), pH 8.0, 1.7% organic matter, a CEC of 11.4 cmol(+) kg^-1^, and a total N content of 1.1 g kg^-1^ (arable layer, beginning of the experiment). As regards other soil nutrients, available P and K were moderately high: 15.48 and 97 mg kg^-1^, respectively; Mg was 247 mg kg^-1^ and Ca 2,619 mg kg^-1^.

The three biofertilizers were compared with untreated controls (CO) in a completely randomized block experimental design (n = 3). Each plot/replicate measured 30 m^2^ (10 × 3 m), and contained 24 rows of plants 12 cm apart. The previous crop was sugar beet. Soil tillage included 30-cm deep plowing followed by two harrowings. Presowing fertilization consisted in incorporating 32 kg ha^-1^ of N, 96 of P_2_O_5_ and 96 of K_2_O into the soil through harrowing. The total amount of N, supplied throughout the crop cycle was 160 kg ha^-1^ as ammonium nitrate. The wheat var. Bologna (SIS, Bologna, Italy) was sown on November 3, 2016, and harvested on June 22, 2017. The crop was protected against weeds, insects and fungal pathogens by specific treatments, following local recommendations. To preserve mycorrhizal fungi's survival, plants were protected from fungal pathogens at the heading stage using active ingredients (i.e., Cyproconazole, Azoxystrobin and Prochloraz) recognized as nonharmful to AMF (Plant Health Care Incorporation, 2009).

### Plant Analysis

Leaf chlorophyll content was monitored twice during the growing cycle, at stem elongation (GS35) (Zadoks et al., 1974) and complete ear emergence (GS59), with a SPAD 502 chlorophyll meter (Konica-Minolta, Hong Kong) on the last fully developed leaf of the main culm (10 plants per plot/replicate). The monitored plants were then harvested to determine fresh and dry shoot biomass (after oven-drying for 36 h at 105°C). Shoot N content was also determined according to the Kjeldahl method, and Ca, K, P, Fe, Mg, and Zn concentrations by inductively coupled plasma-optical emission spectroscopy (ICP-OES) (SPECTRO CirOS Vision EOP, SPECTRO Analytical Instruments GmbH, Kleve, Germany) on 0.4-g microwave acid-digested (7 ml HNO3 65% v/v and 1 ml H_2_O_2_ 30% v/v) samples (Mileston ETHOS 900, Bergamo, Italy) according to the EPA method 3052 (USEPA, 1995). Measurement accuracy was ensured with certified reference materials (ERM-CD281 and BRC-402; JRC-IRMM, Geel, Belgium).

Root colonization across a 1-m-deep soil profile was assessed at full flowering (GS65) on May 3, 2017 using the coring method (n = 3; one core per plot). Soil cores 70 mm in diameter were collected from a central row of the plot at least 1 m from the border. Each core was split into 0.1 m subsamples, which were frozen at –18°C until washing. After separation by a hydraulic sieving-centrifugation device on a 500-µm mesh sieve, the roots were stored in a 15% v/v ethanol solution at 4°C until digitalization. Root images were processed with the KS 300 Rel. 3.0 software (Karl Zeiss, Munich, Germany), using a minimum area of 40 pixels as the threshold for background noise. Root length was determined by the FbL (FiberLength) algorithm, and the mean root diameter as the area-to-length ratio of root objects in each sample (Vamerali et al., 2003). Root length density (RLD) was expressed as cm of root per cm^3^ of soil, and root surface density (RSD) as cm^2^ of root per cm^3^ of soil.

### Grain Yield and Gluten Quality

Wheat grain yield was measured at maturity in the central area of each plot (n = 3) by collecting the grains with a plot combine harvester. The harvest index (grain-to-total shoot weight ratio) was determined in a checking area of 1 m^2^ in each plot, together with the 1,000-kernel weight.

Gluten proteins were analyzed in 30-g seed samples (n = 3) gently milled by six pulses of 10 s each with a Knifetec 1095 (Foss, Hillerod, Denmark). Gliadins, high-molecular-weight glutenins (HMW-GS), and low-molecular-weight glutenin subunits (LWM-GS) were sequentially extracted from 30-mg subsamples, according to Visioli et al. (2018a) protocol. Relative quantification of HMW-GS, LMW-GS, and gliadins was performed spectrophotometrically by colorimetric Bradford assay (Bio-Rad, Hercules, CA, USA) at the 595 nm wavelength, with three technical replicates for each sample. Linear regression between absorbance and protein concentration was obtained through calibration with BSA (Bovine Serum Albumin) standards, and the results expressed as mg g^-1^ of wheat flour. Proteins belonging to the three gluten fractions were also separated by SDS-PAGE and subjected to densitometric analyses to evaluate possible variations in the amounts of single protein subunits, and the results expressed as mg/g^-1^ of flour (Visioli et al., 2018a).

### Rhizosphere Microbial Biomass and Enzymatic Activity

Microbial biomass, enzymatic activity, and bacterial biodiversity were evaluated in the wheat rhizosphere (Rh) and in bulk soil (BS) collected on December 16, 2016. Soil monoliths 0.2 m deep containing the root system of ∼30 plants for each biological replicate were randomly taken from the central area of each plot. Plants were gently extracted from the ground, and after removing most of the soil by shaking, the remaining rhizosphere soil adhering to the roots was carefully collected with a small sterile brush. The rhizosphere soil samples from the 30 plants of each replicate were pooled to obtain one sample of >2 g, which was placed in a sterile Falcon tube and immediately stored at –80°C until analysis. The reference bulk soil sample, without wheat roots, was collected from a 0.2-m-deep profile in uncultivated zones between the plots.

Soil microbial biomass was determined as double-strand DNA (dsDNA) content (Bragato et al., 2016) in 300-mg DW soil samples through DNA extraction with a 0.12 M, pH 8 Na_2_HPO_4_ buffer using bead beating and quantification with PicoGreen reagent, as described by Fornasier et al. (2014). Biomass was expressed as µg dsDNA g^-1^ soil D. W.

Nineteen types of enzymatic activity representing the various nutrient cycles were then determined in treated wheat and compared with untreated controls and bulk soil (n = 3). The enzymes examined were: arylsulfatase (aryS), alpha-glucosidase (alfaG), beta-glucosidase (betaG), alpha-galactosidase (alfa GAL), beta-galactosidase (beta GAL), alpha-mannosidase (alfa_MAN), beta-mannosidase (beta_MAN), glucuronidase (uroni), cellobiohydrolase (cell), xilosidase (xilo), chitinase (chit), leucine-aminopeptidase (leu), tripsin- and papain-like protease (trip), acid phosphomonoesterase (acP), phosphodiesterase (bisP), pirophosphate-phosphodiesterase (piroP), alkaline phosphomonoesterase (alkP), inositol-phosphatase (phytase) (inosit), and nonanoate-esterase (nona). Their activity was measured by a heteromolecular exchange procedure (Cowie et al., 2013) using a solution of lysozyme (3%) and bead beating, as described in Bardelli et al. (2017). All activities were determined in 300-mg DW soil samples and expressed as nmol of MUF (4-methyl-umbelliferyl) min^−1^ g^−1^ soil DW.

### Rhizosphere Microbial Biodiversity Analysis by 16S rRNA Gene Sequencing

DNA was extracted from 0.5-g samples of Rh and BS with the FastDNA^®^ Spin Kit for Soil (MP Biomedicals, Santa Ana, CA, USA) according to the manufacturer's protocols, visualized by electrophoresis on 0.8% (w/v) agarose gels to test for DNA integrity, and quantified by Nanodrop ND1000. 16S rDNA amplification, sequencing and data analysis were performed at GenProbio srl's DNA sequencing facility (www.genprobio.com), according to the protocol described by Visioli et al. (2018b).

Differences between samples in the relative abundances of the taxonomic units were ascertained by a one-way Analysis of Variance (ANOVA). Bacterial taxa with *P*-values <0.05 were selected and identified as the phylotypes or bacterial families that were significantly influenced by the biofertilizers being tested. Pyrosequencing reads were sent to GenBank to obtain their under accession numbers. They are available as bioproject PRJNA388660.

### Statistical Analysis

An ANOVA was carried out on the dataset for all the parameters examined using the Statgraphics Centurion XI software (Adalta, Arezzo, Italy). Separation of means was set at *P ≤* 0.05 with the Newman-Keuls test.

To facilitate interpretation of the whole dataset, a factorial discriminant analysis (MDA, Multigroup Discriminant Analysis with Wilks' lambda and Pillai's trace tests) and a principal component analysis (PCA) were carried out to describe the plant- and microbiologically-related variables. Multivariate data normality was first verified by the Shapiro test. Before analysis, the data were standardized by subtracting the mean and dividing by the standard deviation within each variable. All analyses were performed with MS Excel XLSTAT (Addinsoft, Paris, France).

## Results

### Climatic Conditions During the Trial

The climatic parameters recorded by the local meteorological station (ARPAV, Teolo, Italy) during the field trial showed that the average monthly temperature was quite similar to the 10-year mean (2007–2017), but large differences were found for precipitation. Compared with the historical mean, temperatures were lower in December and January, while rainfall was higher in November but markedly lower for the rest of the cycle, particularly during the winter (**Figure SI 1**). From December to June, overall precipitation was only 170 mm.

### Rhizosphere Soil Microbial Biomass and Biodiversity, and Enzymatic Activity

As expected, the soil microbial biomass in cultivated soil was greater than in bulk soil. There was also greater microbial biomass in the rhizosphere of the inoculated plants than in that of the noninoculated controls, particularly with R-PK, although the difference was not statistically significant (**Table 1**).

**Table 1:** Total microbial biomass (ug dsDNA g^-1^ dry soil; n = 3 ± s.e.) and enzymatic activity (nmol of MUF min^-1^ g^-1^ dry soil; n = 3 ± s.e.) in bulk soil (BS), and in the rhizosphere of *Triticum aestivum* inoculated with biofertilizers (TN, TripleN; R-N, Rhizosum N; R-PK, Rhizosum PK) vs untreated controls (CO).

Enzymatic activity	Treatment									
	BS		CO		TN		R-N		R-PK	
AryS	10.3 ± 2.5	a	12.1 ± 2.2	a	15.2 ± 1.6	a	12.9 ± 0.9	a	12.2 ± 1.2	a
alphaG	1.11 ± 0.2	b	2.11 ± 0.5	ab	3.54 ± 0.5	ab	2.64 ± 1.24	ab	7.35 ± 2.90	a
betaG	4.38 ± 0.8	c	9.11 ± 0.4	b	10.03 ± 0.5	b	11.42 ± 0.6	ab	13.91 ± 1.1	a
alpha GAL	0.46 ± 0.1	b	1.42 ± 0.4	a	1.81 ± 0.2	a	1.77 ± 0.1	a	2.25 ± 0.01	a
beta GAL	1.3 ± 0.3	b	2.7 ± 0.4	a	3.0 ± 0.1	a	2.8 ± 0.1	a	2.8 ± 0.1	a
alpha MAN	0.09 ± 0.02	c	0.26 ± 002	b	0.34 ± 0.03	ab	0.39 ± 0.04	a	0.39 ± 0.03	a
beta MAN	0.13 ± 0.02	b	0.22 ± 0.01	b	0.34 ± 0.01	b	1.19 ± 0.02	a	0.95 ± 0.02	a
Uroni	1.6 ± 0.4	b	3.9 ± 0.2	a	3.8 ± 0.3	a	4.0 ± 0.4	a	4.2 ± 0.4	a
Cell	0.15 ± 0.04	b	0.52 ± 0.1	ab	0.47 ± 0.03	ab	0.77 ± 0.2	a	0.85 ± 0.04	a
Xilo	0.84 ± 0.2	d	1.46 ± 0.1	c	1.76 ± 0.1	bc	2.08 ± 0.2	ab	2.57 ± 0.05	a
Chit	2.3 ± 0.04	b	5.8 ± 0.1	a	5.2 ± 0.8	a	5.2 ± 1.3	a	3.6 ± 0.2	ab
Leu	35.2 ± 6.1	b	64.9 ± 5.9	a	70.1 ± 3.1	a	64.6 ± 3.9	a	70.9 ± 8.3	a
Trip	2.7 ± 0.4	a	4.5 ± 0.5	a	4.8 ± 0.4	a	3.8 ± 0.2	a	4.5 ± 0.7	a
acP	38.4 ± 8.1	a	48.8 ± 4.1	a	54.9 ± 1.5	a	53.1 ± 2.3	a	55.7 ± 2.9	a
bisP	39.8 ± 8.8	a	48.7 ± 7.7	a	54.8 ± 2.8	a	54.4 ± 4.6	a	52.6 ± 4.5	a
piroP	9.4 ± 2.1	a	11.9 ± 2.2	a	13.0 ± 0.9	a	14.9 ± 0.6	a	16.1 ± 2.1	a
alkP	264.4 ± 58	b	357.6 ± 46.5	b	438.5 ± 15.7	a	401.9 ± 27.8	a	375.5 ± 40.8	a
Inosit	0.68 ± 0.2	b	0.93 ± 0.1	ab	1.40 ± 0.1	a	1.15 ± 0.2	ab	1.40 ± 0.1	ab
Nona	68.5 ± 15.2	b	109.6 ± 14.7	ab	101.0 ± 11.4	ab	79.4 ± 13.5	b	131.3 ± 16.8	a
Microbial biomass	16.4 ± 2.2	b	20.5 ± 3.0	ab	21.6 ± 1.2	ab	22.9 ± 1.9	ab	27.4 ± 2.6	a

aryS, arylsulfatase; alfaG, alpha-glucosidase; betaG, beta-glucosidase; alfa GaL, alpha-galactosidase; beta GAL, beta-galactosidase; alpha MAN, alpha-mannosidase; beta MAN, beta-mannosidase; uroni, glucuronidase; cell, cellobiohydrolase; xilom, xylosisase; chit, chitinase; leu, leucine-aminopeptidase; trip, tripsin- and papain-like protease; acP, phosphomonoesterase; bisP, phosphodiesterase; piroP, pirophosphate phosphodiesterase; alkP, alkaline phosphomonoesterase; inosit, inositol phosphatase (Phytase); nona, nonanoate-esterase. Letters indicate statistically significant differences among treatments (Newman-Keuls test, P ≤ 0.05).

**Figure 1:**
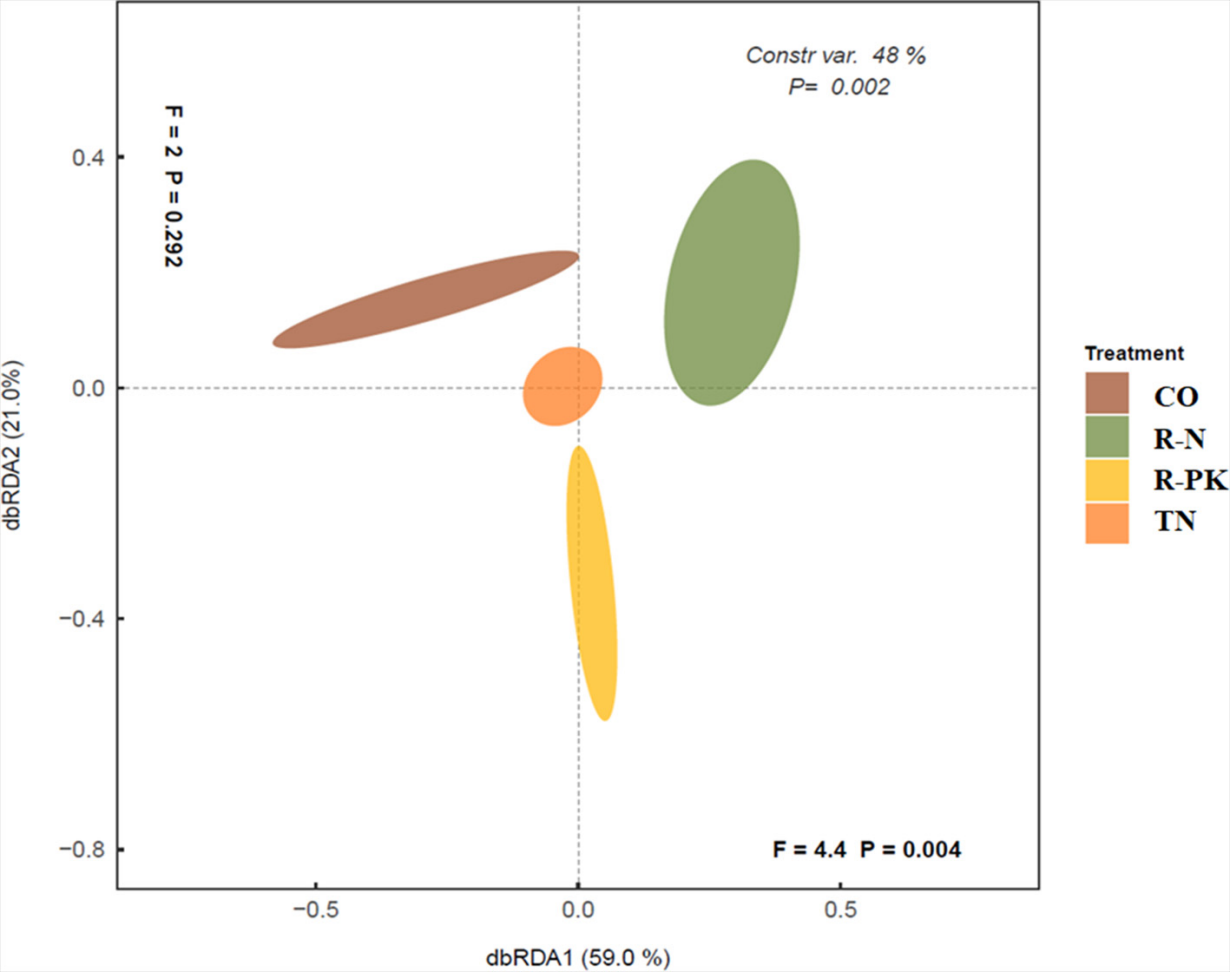
Overall differences among rhizosphere hydrolytic enzyme activities determined by distance-based redundancy analysis (dbRDA) ordination among treatments (TN, TripleN; R-N, Rhizosum N; R-PK, Rhizosum PK) and noninoculated controls (CO). Percentages along the axes show the proportions of dissimilarity captured.

The levels of activity of many of the rhizosphere enzymes investigated here were higher in inoculated plants than in bulk soil, but they were also seldom higher than in the noninoculated controls. The soil microbial enzymatic activity response allowed us to clearly separate treatments by principal coordinate analysis (PCoA) (**Figure 1**), which showed that the R-N and R-PK treatments were more distant from the untreated controls (CO). The two dimensions/variables of db-RDA (distance-based redundancy analysis) explained an overall variability of 81%, mostly attributed to the first (db-RDA1 = 59%, *P =* 0.004) than to the second variable (db-RDA2 = 21%, *P =* 0.292).

Comparison among the rhizospheres of the controls and treated plants showed that the enzymatic activity of beta-glucosidase, α-mannosidase, β;-mannosidase, and xylosidase was significantly higher with R-N and R-PK, i.e., the treatments including plant-aiding fungi, than in the biofertilizer containing only PGP rhizobacteria. The activity of the alkaline phosphomonoesterase was also significantly higher in treated plants, particularly with TN and R-N, and nonanoate-esterase activity was considerably enhanced with R-PK (**Table 1**).

**Figure 2:**
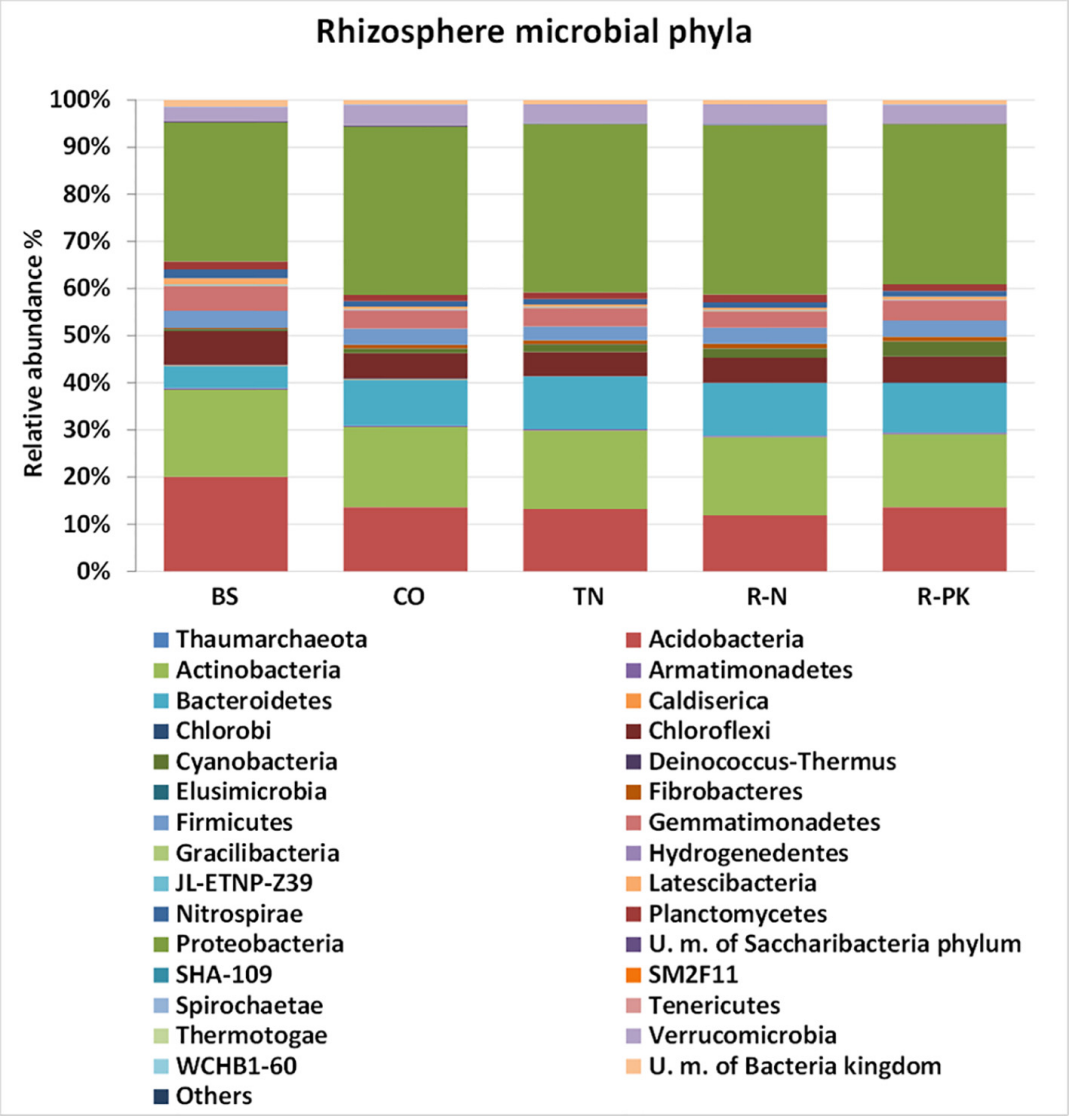
Microbial community composition (%; n = 3 ± s.e.) at the phylum level based on 16S rDNA reads in bulk soil (BS) and the rhizosphere of *Triticum aestivum* inoculated with biofertilizers (TN, TripleN; R-N, Rhizosum N; R-PK, Rhizosum PK) and noninoculated controls (CO) (Newman-Keuls test, *P* ≤ 0.05). Only taxa >0.6% are shown.

**Figure 3:**
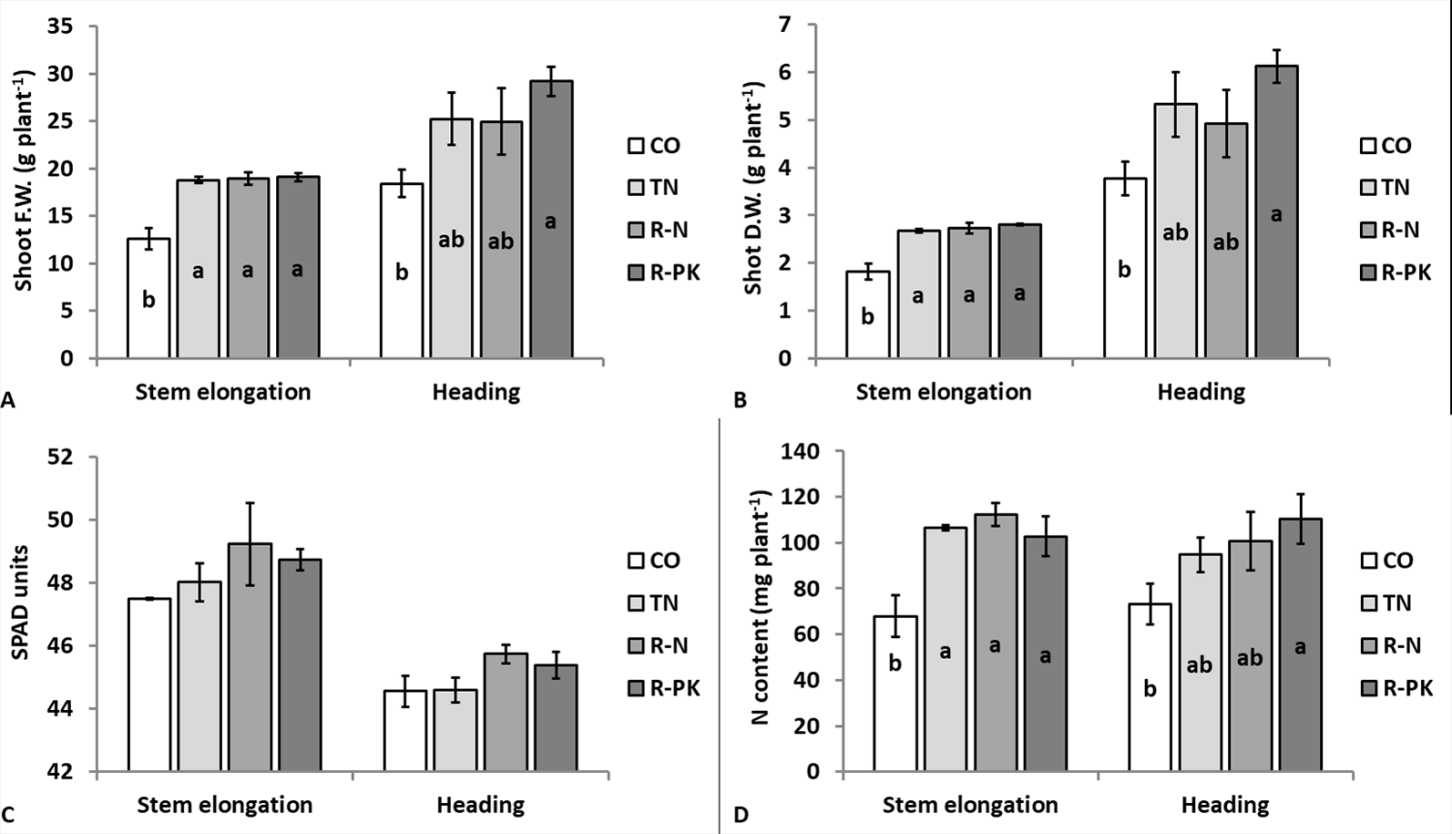
Shoot fresh **(A)** and dry **(B)** weight, leaf chlorophyll content **(C)**, and shoot nitrogen content **(D)** (n = 3 ± s.e.) in *Triticum aestivum* plants inoculated with biofertilizers (TN, TripleN; R-N, Rhizosum N; R-PK, Rhizosum PK) vs untreated controls (CO). Letters indicate statistically significant differences among treatments (Newman-Keuls test, *P* ≤ 0.05).

Regarding bacterial biodiversity in the rhizosphere and the bulk soil, the total number of gene sequences (average of three replicates) ranged from 54,929 to 59,843. Bacterial diversity, measured as OTUs (Operational Taxonomic Units), and the calculated bacterial diversity indices, i.e., the Shannon diversity index and the Chao1 estimator of richness, revealed no significant differences among treatments. The rarefaction curves using the Chao and Shannon indices approached the plateau, indicating that further sequencing would not have resulted in additional OTUs (**Figure SI 2**). Differences in the microbial rhizosphere compositions were detected at both the phylum and family levels (**Figure 2**, **Table SI 1**). The major differences were between bulk soil and the rhizosphere, confirming the significant impact of soil root colonization on microbiota development. In particular, the *Bacteroidetes*, *Cyanobacteria*, *Proteobacteria,* and *Fibrobacteres* phyla were significantly more abundant in the rhizosphere, and *Actinobacteria*, *Acidobacteria*, *Gemmatimonadetes,* and *Chloroflexi* in the bulk soil (**Figure 3**). Some differences were also found between the rhizospheres of the controls and the inoculated plants: seed treatment with R-PK greatly stimulated the abundance of *Cyanobacteria* (+ 24% vs controls), TN the bacteria of the *Flavobacteriaceae* family, which reached 3.34% of the total community vs 1.99% of controls, and R-N the *Planctomycetaceae* family (**Table SI 1**). Compared with controls, R-PK reduced the abundance of the *Verrucomicrobiaceae* family, and R-N the *Gaiellaceae*, although these were present at <1%.

### Wheat Growth and Grain Yield

Wheat plant growth was appreciably enhanced following seed inoculation with biofertilizers, as evidenced by the greater shoot biomass at both the stem elongation (GS35, fifth node detectable) and complete ear emergence (GS59) stages, although on the second observation the R-PK treatment was the only significant (*P* ≤ 0.05) (**Figures 3A, B**). Leaf chlorophyll content of the youngest developed leaf, which decreased with plant aging from stem elongation to heading, was not affected by seed inoculation, although it was slightly improved by R-N and R-PK (**Figure 3C**). However, inoculated plants accumulated a greater amount of nitrogen above ground as a result of both greater biomass and nitrogen concentration, with significant improvements for all treatments at stem elongation, and for R-PK at the heading stage (**Figure 3D**). At this stage, the shoot concentrations of other nutrients (i.e., K, P, Ca, Mg, Fe, and Zn) were generally lower with plant inoculation, but the overall content (uptake) of Ca, K and Zn improved significantly, particularly with R-PK, due to better plant growth (**Table 2**).

**Table 2:** Shoot element (Ca, Calcium; Fe, Iron; K, Potassium; Mg, Magnesium; P, Phosphorus; Zn, Zinc) concentrations and contents (mean ± s.e.; n = 3) at the heading stage of *Triticum aestivum* inoculated with biofertilizers (TN, TripleN; R-N, Rhizosum N; R-PK, Rhizosum PK) vs untreated controls (CO).

Treatment	Concentration (mg kg^-1^)						Content (mg plant^-1^)					
	Ca		Fe		K		Ca		Fe		K	
CO	3200 ± 313	a	262 ± 80.5	a	27768 ± 1395	a	12.1 ± 1.91	b	0.98 ± 0.25	a	105 ± 6.54	b
TN	2476 ± 37	b (-23)	197 ± 24.3	a (-25)	21083 ± 1151	b (-24)	13.2 ± 2.71	ab (+9)	1.04 ± 0.23	a (+6)	112 ± 27.84	ab (+7)
R-N	3004 ± 140	ab (-6)	189 ± 20.7	a (-28)	22211 ± 632	b (-20)	14.8 ± 1.62	ab (+22)	0.93 ± 0.24	a (-6)	109 ± 14.46	ab (+4)
R-PK	2684 ± 100	ab (-16)	210 ± 14.1	a (-20)	19410 ± 578	b (-30)	16.4 ± 0.32	a (+36)	1.28 ± 0.06	a (+30)	119 ± 3.25	a (+14)
	**Mg**		**P**		**Zn**		**Mg**		**P**		**Zn**	
CO	1894 ± 139	a	2764 ± 291	a	29.7 ± 2.04	a	7.14 ± 1.22	a	10.4 ± 0.88	a	0.11 ± 0.01	b
TN	1185 ± 19	b (-37)	2064 ± 122	b (-25)	20.7 ± 0.72	b (-30)	6.31 ± 1.29	a (-12)	11.0 ± 2.76	a (+5)	0.11 ± 0.03	ab (-1)
R-N	1414 ± 65	b (-25)	2171 ± 100	b (-21)	29.5 ± 4.63	ab (-1)	6.95 ± 0.76	a (-3)	10.7 ± 2.06	a (+2)	0.14 ± 0.03	ab (+29)
R-PK	1242 ± 25	b (-34)	1850 ± 170	b (-33)	22.9 ± 2.03	b (-23)	7.60 ± 0.32	a (+7)	11.3 ± 0.51	a (+9)	0.14 ± 0.01	a (+25)

Letters indicate statistically significant differences among treatments within same element (Newman-Keuls test, P ≤ 0.05). In brackets: % variation for each treatment compared with controls.

Wheat yield and its components, i.e., the harvest index (HI), and thousand kernel weight (TKW), were very stable across treatments, with small nonsignificant improvements in the inoculated wheat (*P > *0.05), mainly with R-PK. The average values were: HI = 43%, TKW = 31 g, and yield = 756 g m^-2^.

Destructive root investigations at complete flowering revealed similar root patterns among the inoculated plants and controls in terms of the average whole root profile. However, significant benefits in root length density (RLD) and root area density (RAD) were found in the arable layer with the TN treatment (*P > *0.05). On the other hand, the two biofertilizers containing AMF (i.e., R-N and R-PK) led to slight decreases in root length, area and diameter (**Table 3**).

**Table 3:** Root length density (RLD), root surface density (RSD), and diameter (D) (mean ± s.e.; n = 3) at the flowering stage as averages of different soil layers in *Triticum aestivum* inoculated with biofertilizers (TN, TripleN; R-N, Rhizosum N; R-PK, Rhizosum PK) vs noninoculated controls (CO).

Root parameter	Treatment	Soil depth (m)								
		0-1			0-0.5			0.5-1		
RLD (cm cm^-3^)	CO	3.02	a		4.14	b		1.89	a	
	TN	3.16	a	(+5)	4.68	a	(+13)	1.63	a	(-14)
	R-N	2.72	a	(-10)	3.93	b	(-5)	1.50	a	(-21)
	R-PK	2.73	a	(-9)	3.71	b	(-10)	1.76	a	(-7)
RSD (cm^2^ cm^-3^)	CO	42.0	a		54.9	ab		29.0	a	
	TN	46.2	a	(+10)	66.7	a	(+21)	25.7	a	(-12)
	R-N	37.3	a	(-11)	51.5	b	(-6)	23.1	a	(-21)
	R-PK	37.3	a	(-11)	48.3	b	(-12)	26.4	a	(-9)
D (µm)	CO	293	a		268	a		317	a	
	TN	303	a	(+4)	284	a	(+6)	322	a	(+1)
	R-N	287	a	(-2)	263	a	(-2)	311	a	(-2)
	R-PK	282	a	(-4)	261	a	(-2)	303	a	(-4)

Letters indicate significant differences among treatments within the same parameter and soil depth (Newman-Keuls test, P ≤ 0.05). In brackets: % variation in inoculated plants vs noninoculated controls.

### Effects of Biofertilizers on Gluten Content and Composition

**Figure 4:**
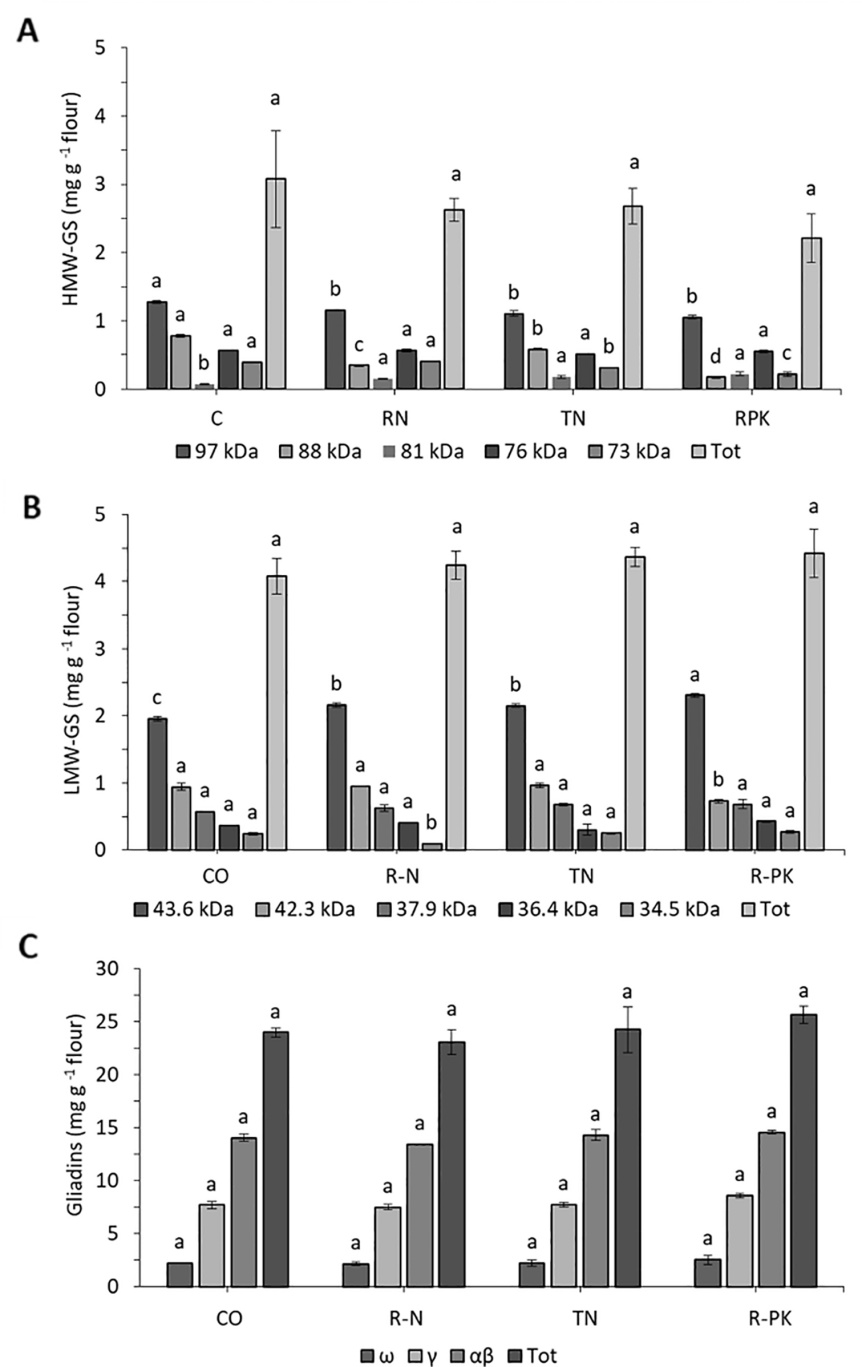
High-molecular-weight glutenin (HMW-GS, **A**), low-molecular-weight glutenin subunits (LMW-GS, **B**), and gliadins **(C)** (mg g^-1^ D. W. flour; n = 3; mean ± s.e.) as represented by different kDa bands revealed with SDS-PAGE in *Triticum aestivum* plants inoculated with biofertilizers (TN, TripleN; R-N, Rhizosum N; R-PK, Rhizosum PK) vs noninoculated controls (CO). Letters indicate significant differences among treatments within the same band (Newman-Keuls test, *P* ≤ 0.05).

Biofertilizers had no significant effects on flour gluten content, which was similar with noninoculated controls, although there was a general slight increase in gliadins and a reduction in glutenins, particularly the high-molecular-weight glutenin subunits (HMW-GS) (**Table 4**). Maximum variations were found with the R-PK treatment, which resulted in a 7% increase in gliadins, and an 8% reduction in glutenins. SDS-PAGE revealed that all the inocula led to a significant increase in the 81 kDa HMW-GS and the 43,6 kDa LMW-GS (**Figures 4A, B**), both playing a key role in technological quality. No significant changes in the composition of the gliadin subunits were observed (**Figure 4C**).

**Table 4:** Total gliadins, low-molecular-weight (LMW-GS) and high-molecular-weight glutenin subunits (HMW-GS), total glutenins (mg g^-1^; n = 3; ± s.e.) and glutenin/gliadin and HMW/LMW ratios in grains of *Triticum aestivum* inoculated with biofertilizers (TN, TripleN; R-N, Rhizosum N; R-PK, Rhizosum PK) vs noninoculated controls (CO).

Treatment	Total gliadins			LMW-glutenins			HMW-glutenins			Total glutenins			Glutenins/Gliadins			HMW/LMW		
	**(mg g^-1^)**			**(mg g^-1^)**			**(mg g^-1^)**			**(mg g^-1^)**								
CO	24.0 ± 0.4	a		4.1 ± 0.2	a		3.1 ± 0.4	a		7.2 ± 0.4	a		0.30 ± 0.02	a		0.75 ± 0.11	a	
TN	24.3 ± 1.3	a	(+1)	4.4 ± 0.1	a	(+7)	2.7 ± 0.2	a	(-13)	7.1 ± 0.2	a	(-3)	0.30 ± 0.02	a		0.62 ± 0.05	a	(-25)
R-N	23.1 ± 0.7	a	(-4)	4.2 ± 0.1	a	(+2)	2.6 ± 0.1	a	(-16)	6.8 ± 0.2	a	(-4)	0.29 ± 0.02	a	(-3)	0.62 ± 0.04	a	(-25)
R-PK	25.7 ± 0.8	a	(+7)	4.4 ± 0.2	a	(+7)	2.2 ± 0.2	a	(-29)	6.6 ± 0.3	a	(-8)	0.26 ± 0.02	a	(-13)	0.50 ± 0.11	a	(-38)

Letters indicate significant differences among treatments within the same parameter (Newman-Keuls test, P ≤ 0.05). In brackets: % variation in inoculated plants vs noninoculated controls.

### Principal Component Analysis

**Figure 5:**
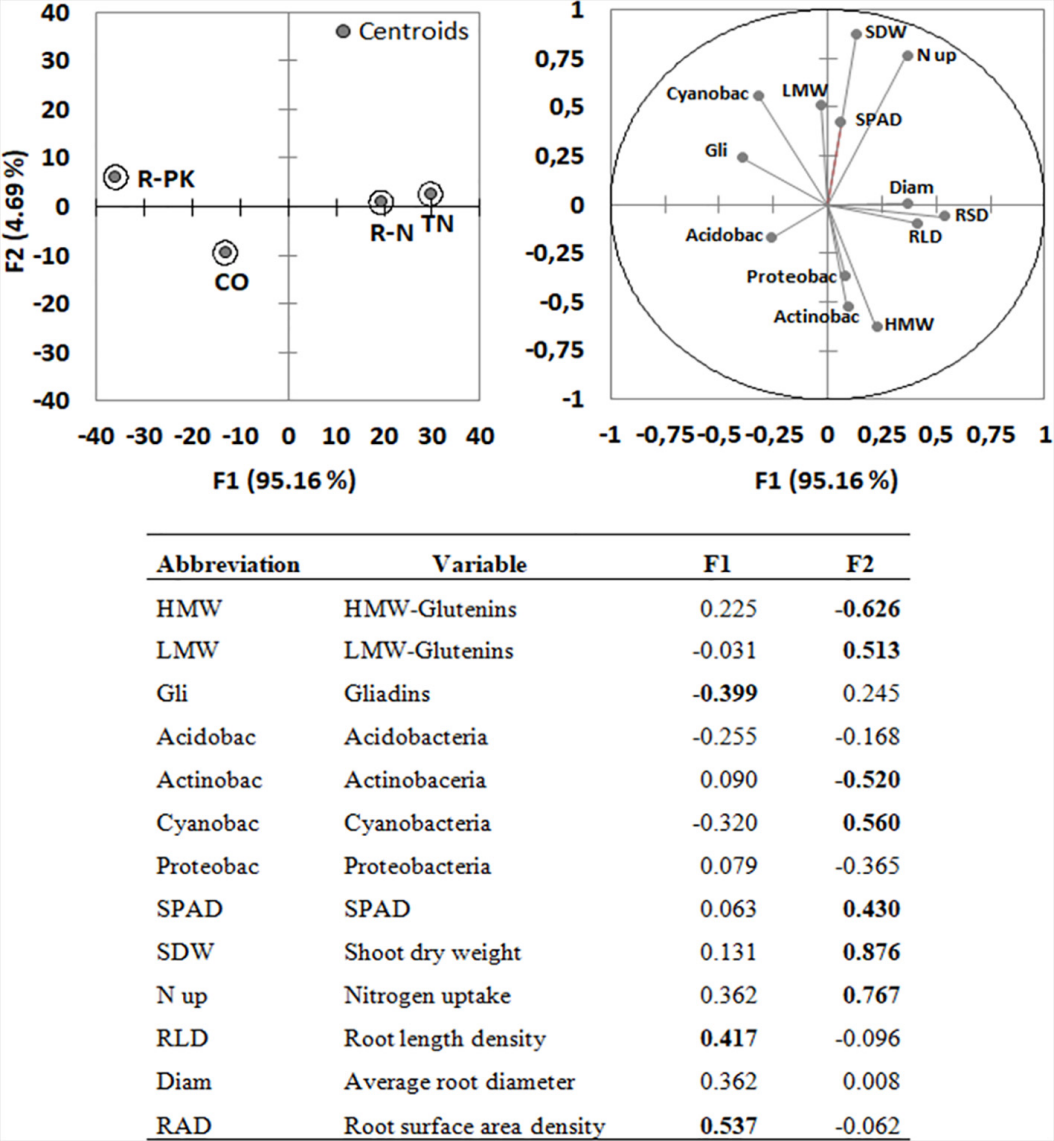
Principal component analysis (PCA; top right) with variable loadings (values > |0.4| in bold; bottom) and discriminant analysis (DA; top left) for *Triticum aestivum* inoculated with biofertilizers (TN, TripleN; R-N, Rhizosum N; R-PK, Rhizosum PK) vs noninoculated controls (CO). Circles in the PCA include 75% of cases.

The PCA identified two synthetic components which explained an overall variability of 99.85%, mostly attributed to the first one (F1 = 95.16%; F2 = 4.69%) (**Figure 5**). Most of the relevant variables (loadings > |0.4|) i.e., gluten subunits, rhizosphere bacterial composition and shoot growth were assigned to F2, while gliadins and root parameters (both length and surface area density) were the most representative ones in F1.

The direction of the vector of each variable indicated generally good correlations among the variables plotted very closely together, i.e., SPAD, shoot biomass, and shoot nitrogen uptake, which were negatively correlated with the abundances of the bacterial phyla *Acidobacteria*, *Proteobacteria,* and *Actinobacteria*. The abundance of *Cyanobacteria* was correlated with LMW-GS and gliadin contents, while HMW-GS content was correlated with root growth and the abundances of *Proteobacteria* and *Actinobacteria*.

The centroid position and cluster separation in the discriminant analysis (**Figure 5**) summarize wheat response to the three biofertilizers, and show that TN and R-N treatments promoted root growth, while R-PK mainly affected gluten composition. In this way, the PCA highlighted the effects on root parameters as the most relevant impact of bacterial inoculation.

## Discussion

Biofertilizers represent a sustainable tool for improving crop yield, as beneficial bacteria and fungi can exert several positive effects on plant nutrition and growth in many crops, including wheat (Basu et al., 2017; Igiehon and Babalola, 2017). Although this is generally clear under laboratory and controlled conditions, the application of plant-aiding microorganisms in open field may be constrained by poor agronomic response. Two of the biofertilizers studied here, i.e., TN composed of a bacteria consortium (*Azospirillum* spp. + *Azoarcus* spp. + *Azorhizobium* spp.), and R-N, a fungal-bacterial consortium (*Rhizophagus irregularis* + *Azotobacter vinelandii*), have already been successfully applied in open field by foliar spraying before stem elongation (Dal Cortivo et al., 2017; Dal Cortivo et al., 2018). In the present study, the biofertilizers were applied as seed inoculants, which is less costly than canopy spraying, with the aim to investigate their potential agronomic effects together with the environmental/ecological impact, which is related to the interaction with the resident microbial community, and the possible mechanisms of interaction with the wheat plants. Using this application method, we expect endophytic PGPR to colonize the root surface and intercellularly colonize the internal plant tissues of different plant organs, and AMF to colonize the plant roots starting with the first rootlet of the germinating seeds, thereby contributing to plant nutrition and growth (Lodewyckx et al., 2002; Smith and Read, 2008; Pagnani et al., 2020). Indeed, efficient colonization of root tissues by PGPR bacteria included in TN, as well as by *Rhizophagus irregularis* contained in R-N and R-PK, has been already documented by electron microscopy (ESEM) in our previous studies (Dal Cortivo et al., 2017; Dal Cortivo et al., 2018). This fits with the appreciable increases in shoot growth and accumulation of minerals, particularly nitrogen, detected across the growing season, although these did not translate into significant increases in wheat yield in our field experiment. The different plant responses may be attributed to the microbial composition of the biofertilizers, as TN mainly stimulated root growth, while R-N and R-PK enhanced the uptake of low-mobile nutrients, such as Ca, K, and Zn, mainly through improved plant growth.

The spectrum of effects of seed inoculation on wheat reported here are similar to previous literature dealing with various growing conditions (Turan et al., 2012; Piccinin et al., 2013; Nadeem et al., 2014). Growth enhancements may be attributed to the N-fixing and nutrient-solubilizing abilities of the applied microorganisms, and to the production of growth-promoting substances, such as IAA (indole-3-acetic acid). PGPR can also modify the level of phytohormones involved in plant senescence through the production of the enzyme ACC-deaminase, and toxins, like rhizobitoxine, which limits ethylene synthesis (Stamenković et al., 2018).

As a result of the associations among a mycorrhizal fungus and P- and K-solubilizing bacteria, such as *Bacillus megaterium* and *Frateuria aurantia* (Elkoca et al., 2010; Velázquez et al., 2016; Ezawa and Saito, 2018), the R-PK treatment was found to engender the best plant growth and nutrient uptake responses, but seldom together with a reduction in nutrient concentrations in the plant tissues, although this is expected where soil fertility is high (Richardson et al., 2009). Mycorrhizal fungi are known to form a highly-developed hyphal network that absorbs nutrients, particularly phosphates, up to several centimeters from the root adsorption zone, often leading to a decrease in fine root length (Liu, 2009; Ezawa and Saito, 2018), as in this trial.

We found a clear positive effect on root growth of inoculation with TN, resulting in greater volumetric root length density in the arable layer. In fact, *Azospirillum* bacteria, included in the formulation of TN, are recognized as stimulating root length and area through the release of auxins, thereby increasing nitrogen and low-mobile nutrients, and water uptake (Bhattacharyya and Jha, 2012). Several studies have reported root growth enhancements, particularly at the early plant stages, but this may be not a stable response in mature plants and across years, as the PGPR-plant association in open field is strongly affected by adverse environmental conditions (e.g., excessive precipitation) after soil/plant inoculation (Basaglia et al., 2003; Dal Cortivo et al., 2017). However, this experiment confirms that soil conditions and fertility were probably favorable to the onset of the plant-PGPR signals that precede colonization in competition with the resident microbiome (Videira e Castro et al., 2016), allowing wheat to benefit in terms of root growth.

Wheat seed inoculation also had a clear positive impact on the rhizosphere microbiome, at least relatively soon after sowing (about 6 weeks). There was a general increase in total microbial biomass and some soil enzymatic activities, demonstrating enhanced microbial metabolism, mainly when the inoculum contained both plant-aiding bacteria and AMF. The increased enzymatic activities included beta-glucosidase, which hydrolyzes cellobiose to free glucose (Zang et al., 2018), alfa-mannosidase, beta-mannosidase, and xylosidase, which is produced by both endophytic bacteria and fungi, and which hydrolyzes mannans and xylans as the main components of lignified organic materials together with cellulose and lignin (Nankai et al., 2002; Robl et al., 2013). Alkaline nonophosphoesterase, a key enzyme for organic P degradation into inorganic phosphate available for plant uptake, particularly under P-limiting conditions (Acuña et al., 2016), was also generally upregulated by inoculation. Nonanoate-esterase activity was increased significantly by the R-PK treatment, although other contributing enzymes act on ester bonds, including esterases and proteases.

An important issue to be considered in plant/soil inoculation is the possible impact on the resident microbiome. In our study, there was a considerable higher bacterial biodiversity in the rhizosphere compared with bulk soil, confirming the essential role played by root presence in the soil microbiome. On the other hand, we found small differences between the rhizospheres of the treated plants vs the noninoculated controls in terms of bacterial biodiversity, suggesting that all the inocula applications studied here are safe. Nevertheless, inoculation modified the abundances of specific rhizosphere bacteria phyla. Interestingly, the R-PK inoculum highly stimulated the *Cyanobacteria*, which are known to form associations with wheat roots to alleviate nitrogen deficiency and enhance the rhizosphere microbial biomass (Karthikeyan et al., 2007). As *Cyanobacteria* are also able to produce plant growth-promoting substances, it has been suggested they be included as inoculants for rice and maize (Prasanna et al., 2016). Bacteria belonging to the *Flavobacteriaceae* family, which were stimulated by the TN treatment, may also be beneficial for wheat in this experiment, as they proved to have important ecological functions contributing to organic matter turnover and pesticide decomposition (Wolińska et al., 2017), which in part helps explain the increase in some soil enzymatic activities (**Table 1**).

The main agronomic result obtained in this study was the greater nitrogen uptake in inoculated plants, although this did not translate into significant gains in grain yield, and no effects on grain quality in terms of gluten content were observed, in contrast with the findings of some authors which tested the effects of PGPR inoculation in ancient *Triticum* varieties (Pagnani et al., 2020). Accumulation of gluten proteins is a complex process involving spatial and temporal regulation. Environmental conditions, such as heat and drought stress, as well as the dose and application timing of nitrogen fertilizers can affect significant changes in gluten composition (Flagella et al., 2010; Visioli et al., 2018a). However, very little information is available on possible changes in gluten protein composition in response to biofertilizer application. Stępień and Wojtkowiak (2013) showed that gluten content, particularly of HMW-GS and LMW-GS, increased in spring and winter wheat cultivars with organic fertilizers, regardless of the addition of effective microorganisms. A novel finding in this study is the significant upregulation of certain LMW-GS and HMW-GS, the latter being polymeric proteins involved in dough strength and elasticity (Sissons, 2008). In particular, the 81 kDa HMW-GS is the Bx subunit codified by the locus *Glu-B1* closely correlated with the technological quality of flour (Sissons, 2008), and the LMW-2 asset, in which the LMW-GS with the highest molecular weight (~44 kDa) is the most abundant subunit of this class (D'Ovidio and Masci, 2004) and is generally upregulated by N supply (Visioli et al., 2018a). Hence, the upregulation of both 81 kDa HMW-GS and 43.6 kDa LMW-GS with all the biofertilizers tested, particularly R-PK, may be attributed to the N-fixing and nutrient-solubilizing contribution of the applied microorganisms or the changes induced in the bacteria groups during grain filling.

## Conclusion

This study has shown that bacteria or bacteria-AMF consortia tested can be safely applied as seed inocula, as they did not alter the bacterial taxa associated with wheat roots allowing the resident microbial biodiversity to be preserved. The benefits of seed inoculation included enhancement of the rhizosphere microbial biomass and of the activity of enzymes involved in organic matter decomposition and nutrient release, especially when the biofertilizer contains the AMF *Rhizophagus irregularis*. We confirmed the importance of diazotrophic bacteria (i.e., *Azoarcus*, *Azospirillum*, *Azorhizobium*) in enhancing plant nitrogen nutrition and root growth. Despite with moderate effects, the biofertilizers tested here are also expected to alleviate nutrient deficiency, particularly P and Fe, which is of great importance in the worldwide spread alkaline soils, like in our site. Seed inoculation is therefore suitable for aiding the fertilization of wheat, and possibly of other cereals in organic agriculture, and may also provide beneficial environmental effects in reducing N losses.

We did not find any improvements in grain yield, although significant agronomic benefits in poor soil, low-input agriculture or under abiotic stress conditions cannot be excluded. A new finding was the increase in gluten quality due to upregulation of specific gluten protein subunits, which can be exploited in the wheat food chain.

Currently, the most suitable option seems to be the association between bacteria and AMF, but future research is required to gain further specific insights into biofertilizer composition in order to exploit the synergistic action of various bacteria and mycorrhizal fungi, and understand their behavior under reduced soil resource availability.

## Data Availability Statement

The sequencing data generated by this study can be found in the NCBI under accession number bioproject PRJNA388660.

## Author Contributions

TV conceived and designed the experiment, and revised the MS. CC and MF carried out the experiment and analyzed the data. GV and ML carried out gluten protein analyses and metagenomics analyses, and FF performed soil enzymatic activities analysis. GB performed the statistical analysis of data. CC wrote the first draft of the paper. AP contributed to improve the *Introduction* and the *Discussion* sections. All authors contributed to interpreting and discussing the results, and read and approved the final version of the manuscript.

## Conflict of Interest

The authors declare that the research was conducted in the absence of any commercial or financial relationships that could be construed as a potential conflict of interest.
