# Supplementary material for: Effects of Seed-Applied Biofertilizers on Rhizosphere Biodiversity and Growth of Common Wheat (Triticum aestivum L.) in the Field
Source: Front Plant Sci. 2020 Feb 26;11:72. doi: 10.3389/fpls.2020.00072 (PMC7054350; doi:10.3389/fpls.2020.00072)
Supplement: Supplementary file 1 [file DataSheet_1.docx]

Supplementary Material

Table SI 1. Microbial community composition (%; n = 3 ± s.e.) at the family level based on 16S rDNA reads in bulk soil (BS) and the rhizosphere of *Triticum aestivum* inoculated with biofertilizers (TN, R-N, R-PK) and non-inoculated controls (CO) (Newman-Keuls test, *P* ≤0.05). Only families >0.10% are shown.

| **Taxonomy** | **BS** | | **CO** | | **TN** | | **R-N** | | **R-PK** | |
| --- | --- | --- | --- | --- | --- | --- | --- | --- | --- | --- |
| *U. m. of Subgroup 17 order (Acidobacteria phylum)* | 0.71 | a | 0.46 | b | 0.40 | b | 0.37 | b | 0.40 | b |
| *U. m. of Subgroup 3 order (Acidobacteria phylum)* | 0.76 | a | 0.50 | b | 0.53 | b | 0.57 | b | 0.53 | b |
| *U. m. of Subgroup 4 order (Acidobacteria phylum)* | 0.60 | c | 0.85 | a | 0.84 | a | 0.62 | bc | 0.80 | ab |
| *U. m. of Subgroup 5 order (Acidobacteria phylum)* | 0.62 | a | 0.29 | b | 0.27 | b | 0.31 | b | 0.29 | b |
| *U. m. of Subgroup 6 order (Acidobacteria phylum)* | 10.32 | a | 6.91 | b | 6.38 | b | 5.78 | b | 6.36 | b |
| *U. m. of Subgroup 7 order (Acidobacteria phylum)* | 1.28 | a | 0.89 | a | 1.02 | a | 0.93 | a | 1.13 | a |
| *U. m. of Acidobacteria phylum* | 0.52 | a | 0.20 | b | 0.19 | b | 0.18 | b | 0.20 | b |
| *U. m. of Acidimicrobiales order* | 0.71 | a | 0.52 | b | 0.55 | b | 0.56 | b | 0.51 | b |
| *Gaiellaceae* | 1.33 | a | 0.97 | b | 0.91 | bc | 0.78 | c | 0.94 | bc |
| *U. m. of Gaiellales order* | 2.37 | a | 1.57 | b | 1.51 | bc | 1.20 | c | 1.59 | b |
| *Microbacteriaceae* | 0.59 | a | 0.87 | a | 0.86 | a | 0.82 | a | 0.62 | a |
| *Micrococcaceae* | 1.33 | b | 2.30 | a | 2.40 | a | 2.51 | a | 2.05 | ab |
| *Micromonosporaceae* | 0.63 | a | 0.64 | a | 0.88 | a | 0.56 | a | 0.44 | a |
| *Nocardioidaceae* | 1.76 | a | 1.92 | a | 1.88 | a | 1.75 | a | 1.88 | a |
| *Propionibacteriaceae* | 1.04 | a | 0.38 | b | 0.41 | b | 0.89 | ab | 0.48 | ab |
| *Streptomycetaceae* | 1.22 | a | 1.60 | a | 1.41 | a | 1.70 | a | 1.27 | a |
| *U. m. of Actinobacteria phylum* | 1.28 | a | 0.91 | b | 0.89 | bc | 0.75 | c | 0.88 | bc |
| *Cyclobacteriaceae* | 0.01 | a | 0.56 | a | 0.15 | a | 0.17 | a | 0.05 | a |
| *Cytophagaceae* | 2.13 | b | 2.89 | a | 2.82 | a | 3.26 | a | 3.12 | a |
| *Flavobacteriaceae* | 0.16 | b | 1.99 | b | 3.34 | a | 2.42 | ab | 2.60 | ab |
| *Chitinophagaceae* | 1.64 | b | 2.55 | a | 2.73 | a | 3.04 | a | 2.72 | a |
| *Eubacterium env.OPS 17* | 0.20 | b | 0.39 | a | 0.45 | a | 0.49 | a | 0.36 | a |
| *Sphingobacteriaceae* | 0.08 | b | 0.61 | a | 0.71 | a | 0.70 | a | 0.71 | a |
| *Anaerolineaceae* | 1.65 | a | 1.13 | b | 1.06 | b | 1.17 | b | 1.18 | b |
| *Caldilineaceae* | 0.59 | a | 0.45 | b | 0.43 | b | 0.46 | b | 0.45 | b |
| *U. m. of JG30-KF-CM45 order* | 0.61 | a | 0.56 | a | 0.52 | a | 0.55 | a | 0.52 | a |
| *U. m. of Chloroflexi phylum* | 2.27 | a | 1.58 | b | 1.49 | b | 1.40 | b | 1.51 | b |
| *U. m. of Cyanobacteria order* | 0.18 | b | 0.37 | a | 0.48 | a | 0.48 | a | 0.54 | a |
| *Fibrobacteraceae* | 0.19 | b | 0.80 | a | 0.85 | a | 1.01 | a | 0.96 | a |
| *Bacillaceae* | 1.73 | a | 1.93 | a | 1.47 | a | 1.80 | a | 1.82 | a |
| *Paenibacillaceae* | 0.42 | a | 0.55 | a | 0.59 | a | 0.58 | a | 0.53 | a |
| *U. m. of AT425-EubC11 terrestrial group order* | 0.25 | b | 0.43 | ab | 0.43 | ab | 0.34 | ab | 0.58 | a |
| *Gemmatimonadaceae* | 3.68 | a | 2.39 | b | 2.45 | b | 2.19 | b | 2.65 | b |
| *U. m. of S0134 terrestrial group order* | 1.17 | a | 0.83 | b | 0.85 | b | 0.84 | b | 0.85 | b |
| *U. m. of Latescibacteria phylum* | 1.36 | a | 0.57 | b | 0.53 | b | 0.50 | b | 0.62 | b |
| *U. m. of 0319-6A21 order* | 0.83 | a | 0.48 | b | 0.44 | b | 0.44 | b | 0.46 | b |
| *Nitrospiraceae* | 1.04 | a | 0.74 | b | 0.70 | b | 0.69 | b | 0.77 | b |
| *Planctomycetaceae* | 0.86 | ab | 0.65 | c | 0.68 | c | 0.92 | a | 0.69 | bc |
| *U. m. of WD2101 soil group order* | 0.50 | a | 0.45 | a | 0.56 | a | 0.52 | a | 0.52 | a |
| *Bdellovibrionaceae* | 0.33 | b | 0.52 | a | 0.52 | a | 0.48 | a | 0.51 | a |
| *Comamonadaceae* | 1.19 | b | 2.63 | a | 2.77 | a | 2.36 | a | 2.38 | a |
| *Oxalobacteraceae* | 0.39 | b | 4.17 | a | 4.98 | a | 4.41 | a | 4.97 | a |
| *Caulobacteraceae* | 0.17 | c | 0.56 | ab | 0.61 | a | 0.38 | bc | 0.42 | ab |
| *Cellvibrionaceae* | 0.06 | b | 0.50 | a | 0.35 | a | 0.47 | a | 0.37 | a |
| *Nitrospinaceae* | 1.11 | a | 0.74 | b | 0.68 | b | 0.70 | b | 0.74 | b |
| *Enterobacteriaceae* | 0.14 | a | 0.04 | a | 0.03 | a | 2.16 | a | 0.18 | a |
| *U. m. of GR-WP33-30 order* | 1.48 | a | 0.97 | b | 0.85 | b | 0.88 | b | 1.01 | b |
| *U.m. of BIrii41 order* | 0.38 | a | 0.45 | a | 0.32 | a | 0.42 | a | 0.37 | a |
| *Haliangiaceae* | 0.75 | a | 0.56 | b | 0.53 | b | 0.44 | b | 0.53 | b |
| *Polyangiaceae* | 0.30 | a | 0.45 | a | 0.47 | a | 0.38 | a | 0.44 | a |
| *Sandaracinaceae* | 0.25 | a | 0.50 | a | 0.41 | a | 0.44 | a | 0.35 | a |
| *Nitrosomonadaceae* | 2.44 | a | 1.44 | b | 1.61 | b | 1.43 | b | 1.69 | b |
| *U. m. of Proteobacteria phylum* | 0.83 | a | 0.57 | b | 0.52 | b | 0.51 | b | 0.53 | b |
| *Pseudomonadaceae* | 0.31 | b | 1.32 | ab | 2.04 | a | 2.06 | a | 0.97 | ab |
| *Bradyrhizobiaceae* | 0.63 | a | 0.60 | a | 0.57 | a | 0.53 | a | 0.51 | a |
| *Hyphomicrobiaceae* | 1.27 | ab | 1.36 | a | 1.13 | ab | 1.07 | b | 1.10 | b |
| *Methylobacteriaceae* | 0.55 | a | 0.66 | a | 0.63 | a | 0.76 | a | 0.69 | a |
| *Rhizobiaceae* | 0.51 | b | 1.31 | a | 1.17 | a | 1.20 | a | 1.04 | a |
| *U. m. of Rhizobiales order* | 0.54 | a | 0.77 | a | 0.58 | a | 0.55 | a | 0.58 | a |
| *Rhodobiaceae* | 0.49 | a | 0.38 | ab | 0.31 | b | 0.35 | b | 0.36 | ab |
| *Xanthobacteraceae* | 0.73 | a | 0.50 | b | 0.44 | b | 0.43 | b | 0.47 | b |
| *Rhodocyclaceae* | 0.13 | b | 0.59 | a | 0.37 | ab | 0.42 | ab | 0.20 | b |
| *U.m. of MSB-1E8 order* | 0.82 | a | 0.60 | b | 0.53 | b | 0.47 | b | 0.60 | b |
| *Rhodospirillaceae* | 1.15 | a | 0.99 | ab | 0.96 | ab | 0.87 | b | 0.98 | ab |
| *U. m. of Rhodospirillales order* | 1.23 | a | 0.92 | b | 0.83 | bc | 0.77 | c | 0.83 | bc |
| *Sphingomonadaceae* | 1.29 | b | 2.09 | a | 2.07 | a | 1.88 | ab | 2.13 | a |
| *U. m. of TRA3-20 order* | 0.65 | a | 0.21 | b | 0.23 | b | 0.25 | b | 0.21 | b |
| *Xanthomonadaceae* | 0.87 | b | 1.46 | a | 1.64 | a | 1.43 | a | 1.44 | a |
| *U. m. of Xanthomonadales order* | 2.78 | a | 1.76 | b | 1.67 | b | 1.70 | b | 1.59 | b |
| *Chthoniobacteraceae* | 0.31 | b | 0.63 | a | 0.52 | ab | 0.61 | a | 0.51 | ab |
| *Opitutaceae* | 0.43 | b | 1.16 | a | 0.93 | ab | 0.88 | ab | 0.85 | ab |
| *U. m. of Verrucomicrobia phylum* | 0.60 | a | 0.45 | bc | 0.43 | bc | 0.40 | c | 0.53 | ab |
| *U. m. of OPB35 soil group class* | 1.00 | a | 0.66 | b | 0.63 | b | 0.64 | b | 0.73 | b |
| *Verrucomicrobiaceae* | 0.31 | c | 1.10 | ab | 0.99 | ab | 1.17 | a | 0.84 | b |
| *U. m. of Bacteria kingdom* | 1.39 | a | 0.95 | b | 0.92 | b | 0.89 | b | 0.94 | b |
| *Others* | 20.37 | a | 18.23 | b | 17.63 | b | 18.15 | b | 18.13 | b |

U. m. = Unclassified member

Figure SI 1. Monthly mean temperatures (A) and rainfall (B) across the crop cycle of wheat at the experimental farm of the University of Padua (Legnaro, Italy).

Figure SI 2. Average rarefaction curves generated for 16S rRNA gene sequences using the Chao1 (A) and Shannon indices (B).
